# Clinical Efficacy of Two Novel, Differentially Administered (IM, ID) Vaccines against *Mycoplasma hyopneumoniae* and PCV2 in Swine under Field Conditions

**DOI:** 10.3390/ani12243467

**Published:** 2022-12-08

**Authors:** Wolfgang Sipos, Sabine Sipos

**Affiliations:** 1 Clinical Department for Farm Animals and Herd Management, University of Veterinary Medicine Vienna, 1210 Vienna, Austria; 2 Veterinary Practice Schwertfegen, 3040 Neulengbach, Austria

**Keywords:** enzootic pneumonia, CLP, lung score, intradermal vaccine, slaughterhouse

## Abstract

**Simple Summary:**

*Mycoplasma hyopneumoniae* (*M.hp.*) and porcine circovirus type 2 (PCV2) are among the most prominent inducers of the porcine respiratory disease complex (PRDC). Therefore, pharmaceutical companies do their best to improve vaccines against these two pathogens for piglets. This field study evaluates the efficacy of two novel vaccines (Hyogen^®^ + Circovac^®^ vs. MHyoSphere^®^PCV ID) with respect to clinically relevant parameters, i.e., average daily weight gain, coughing/sneezing index, losses due to morbidity/mortality, and lung scoring data, in a fattening farm in Lower Austria. Both vaccines proved very suitable measures for controlling disease.

**Abstract:**

Enzootic pneumonia (EP) of pigs is caused by *Mycoplasma hyopneumoniae* (*M.hp.*), which is, together with the porcine circovirus type 2 (PCV2), among the most prominent inducers of the porcine respiratory disease complex (PRDC). Therefore, vaccination of piglets against *M.hp.* and PCV2 is crucial in the fight against pulmonary infections. In this field study, we tested the clinical efficacy of two novel vaccines, one delivered IM (Hyogen^®^ + Circovac^®^) and the other ID (MHyo-Sphere^®^PCV ID), on a fattening farm in Lower Austria with a history of still ongoing EP. Average daily weight gain, coughing/sneezing index, losses due to morbidity/mortality, and lung scoring data at slaughter by means of CLP (Ceva Lung Program) were recorded for three consecutive fattening cohorts to achieve a powerful number of animals, one half each vaccinated with the IM vaccine and the other half with the ID vaccine (n = 659 in total). No statistically significant differences could be observed between the two vaccination groups for the parameters investigated, but the total median EP score, which categorizes pulmonary lesions due to infection by *M.hp.* with a theoretical range of 0–28, was lowered from initially 1.9 to 1.0, indicating that both vaccines proved very suitable measures in the fight against EP.

## 1. Introduction

*Mycoplasma hyopneumoniae* (*M.hp.*) is considered as a primary pathogen of the porcine respiratory system causing enzootic pneumonia (EP) and is thus, together with porcine circovirus type 2 (PCV2), one of the two main drivers of the porcine respiratory disease complex (PRDC) [1,2]. Focal, sharply bordered and deeply dark-red-colored areas of pneumonic tissue, especially in the cranioventral pulmonary regions, are characteristic for EP [3], which seldom occurs as an isolated pathological entity due to the prevalence of numerous respiratory pathogens and high pathogen pressure in many herds [4]. Among the respiratory pathogens regularly contributing to PRDC in Austria, but also in many other countries, are the PRRSV (porcine reproductive and respiratory syndrome virus), *Streptococcus suis* and *Actinobacillus pleuropneumoniae* [3,5].

Pathogenesis of EP is based on respiratory ciliostasis, loss of the cilia, and direct toxic harm to the respiratory epithelium. *M.hp.* isolates from different herds and also within herds are genetically highly heterogeneous. Thus, one may believe from a theoretical point of view, that vaccines could not work well and antibiotics, such as tetracyclines and macrolides, among others, should be the first choice to control and treat EP. However, antibiotics are neither able to eliminate *M.hp.*, nor restore lung lesions [6]. Additionally, the enormous use of antibiotics in recent years has led to a rise in antibiotic resistances, which has important drawbacks for animal and human health and thus is under intense discussion these days. Since start of vaccinations against porcine circovirus type 2 (PCV2), this pathogen rarely leads to pronounced clinical signs. This was different before the routine introduction of vaccines approx. 17 years ago when the postweaning multisystemic wasting syndrome (PMWS) and porcine dermatitis and nephropathy syndrome (PDNS), whose PCV2-associated pathogenesis had been unraveled not long before that time, were relatively frequent [7,8,9].

Contrary to the above-mentioned concerns, vaccination against *M.hp.* has proven to be a very effective measure against EP and severe outbreaks of PRDC [10]. Although clinical signs of an infection by *M.hp.* usually occur sometime later in a weaner’s or fattener’s life, piglets occupy key positions in epidemiological considerations as animals that become infected with *M.hp.* within the suckling period are considered to be the main disease promotors, besides gilts introduced in piglet production units [11].

Traditionally, two-shot vaccine formulations, administered in the first and third week of life, were implemented for EP control [12]. In more recent times, one-shot formulations, mostly administered around week 3, progressively displaced the older vaccines and for good reason. The need for only one single handling of piglets for vaccination is more cost effective and less stressful for the animals but demands for novel formulations (adjuvants) to overrule the draw-back of a single vs. a dual vaccination, which acts as booster. Additionally, modern vaccines increasingly are designed bivalent as they also include the second main driver of PRDC, namely PCV2. Finally, progress is also made concerning adjuvants, which not only have to be effective in promoting a strong specific immune response, but should also exhibit a high tissue compatibility, i.e., they should not induce an exaggerated inflammatory tissue response [13]. 

Another development concerns the route of administration. Vaccines typically are injected subcutaneously (SC) or intramuscularly (IM). A rising number of companies now provides formulations and equipment for intradermal (ID) vaccinations, often with “smart” injection systems, that inform the user (veterinarian, farmer) about the number of doses applied and the number remaining in the vial as well as a series of other data. The needleless administration is certainly less stressful and painful and also is advantageous from an epidemiologic point of view, as no needles are in use that could transmit pathogens. On the other hand, it cannot be excluded under field conditions that occasionally some part of the injection volume is blown out without entering the dermis, although the respective companies try hard to develop systems that (should) eject only in case of a fitting contact and angle to the animal’s skin.

The aim of this study was to compare the efficacy of a novel, but already highly accepted one-shot IM vaccine against *M.hp.* (combined with a second one against PCV2 according to the manufacturer’s instructions) with that of a just released ID vaccine by assessment of clinical signs, performance, and macroscopic lung lesions at slaughter. Thus, this study was also intended to test the ID vaccine for non-inferiority. The IM vaccine is based on the 2940 *M.hp.* strain + inactivated PCV2 and Imuvant™ (Ceva, Germany), a combination of light liquid paraffin O/W and *E. coli* J5 lipopolysaccharide, as an adjuvant. The ID vaccine uses a physically inactivated, recombinant *M.hp.^cpPCV2^*-strain (Nexhyon), which expresses the PCV2-capsid protein. Both vaccines are approved for vaccination of piglets starting from week 3.

## 2. Materials and Methods

### 2.1. Farm History

The study was performed on a PRRS-free fattening farm in Lower Austria. Every 4 weeks a group of 330 piglets from only one origin (based on regular producer-fattener cooperation) at an age of approx. 21 days is delivered. The feed is delivered via spot-mix. Before start of the study, piglets had been vaccinated against *M.hp.* and PCV2 using a two-shot vaccine (Suvaxyn^®^ Circo + MH RTU, Zoetis, Germany) on the production farm at week 1 and after weaning, i.e., immediately before transport to the fattening farm. As clinical signs of EP, i.e., recurrent dry coughing, increased despite additional oral tylosin-tartrate administrations (4 times within 14 months before the start of the study) and EP was confirmed by (I) doing lung checks at slaughter by means of CLP (see Section 2.3), as well as by histopathology, revealing EP-typical lesions (peribronchial pneumonia), and (II) positive PCR results for *M.hp.* (as well as for PCV2 out of lung tissue). This was evidence of either a non-functioning vaccination management by the producer and/or use of a non-valid vaccine. Thus, we decided to change the vaccination regime by vaccinating the piglets on day 3 after delivery and by changing the vaccine. Therefore, this time point of change was ideal for simultaneously testing the efficacy and comparing the two new vaccines. Group A was vaccinated with Hyogen^®^ + Circovac^®^ (Ceva, Germany) delivered IM as a combination, and group B with MHyoSphere^®^PCV ID (Hipra, Spain) administered with the needleless Hipradermic^®^3.0 (Hipra, Spain) device.

As piglets not only suffered from EP but also from enteritis + ileitis, arthritides, and umbilical infections already before start of the trial, animals were subject to a constitutive initial treatment with colistin, amoxicillin, and tylosin-phosphate, which acts only in the GI-tract and thus has no impact on mycoplasmas. This antibiotic treatment helped in reducing the prevalence of the mentioned pathologies to an absolute minimum throughout the whole fattening period.

### 2.2. Study Design

The trial started in January 2022, included three consecutive batches to achieve a powerful number of animals, and ended in October 2022 with the last lung score at slaughter. In summary, 659 healthy piglets were individually ear-tagged, weighed (6.82 kg in the mean), and vaccinated at day 24. Each room consisted of six pens, with three on each side of a central corridor. All three pens of one side were given vaccine A (IM) and of the other side vaccine B (ID), meaning that within each room half of the animals were vaccinated with one and the other half with the other vaccine to exclude systemic errors due to different pathogen pressures in different rooms. In cohort 1, 111 animals were included in group A and 108 in group B and 110 pigs were included in each group in cohorts 2 and 3. When allocating the animals to groups, only the medium-sized animals were chosen and the smallest and biggest ones were excluded in order to achieve a homogeneous distribution of body weights.

Cough/sneezing monitoring was performed by only one investigator every two weeks until the end of the fattening period. Therefore, the number of coughs/sneezes was counted during a period of two minutes and the respective index was obtained by dividing the number of coughs/sneezes by the number of observation time points. The counts were adjusted to the number of pigs in each pen. The weights were individually measured again at week 15 and shortly before slaughter at week 23. Additionally, average daily weight gain (ADWG) from inclusion to slaughter and also overall mortality rate were documented.

### 2.3. Lung Checks

The lungs were scored blindly (one investigator stood at the start of the line and noted the order of the ear tag numbers and the second one performed the scoring without knowing the pig’s identity; the tag numbers were assigned to the scores later) at the slaughterhouse according to the CLP (Ceva Lung Program), a methodology widely used in the field in Austria [12,14,15,16], with 221 pigs in group A and 214 pigs in group B in total. 

In short, lung lesions are categorized first according to a modified Madec–Kobisch Score and a delineated EP (enzootic pneumonia) score, thus considering lesions predominantly caused by *M.hp.*, with a theoretically possible range of values between 0 and 28 and every value > 0 listed as BP (bronchopneumonia). Second, dorsocaudal pleurisies, which are judged by means of the SPES (Slaughterhouse Pleurisy Evaluation System)-method and given as a delineated APP index, which is the averaged SPES score within a possible range between 0 and 4, are recorded. Third, each lung was inspected for the presence of cranioventral pleurisy (CP) without describing the extension of the lesion and, finally, each lung was inspected for the presence of scars.

### 2.4. Statistics

Statistical differences between metric data were analyzed by an unpaired T-test after checking for normal distribution by means of the Anderson-Darling-test (with *p* > 0.05 meaning normal distribution) and by the Mann-Whitney-U-test if not distributed normally. For dichotomic data the Chi^2^-test was used in Excel. *P*-values > 0.05 were not considered statistically significant, but as trends if they were < 0.1.

## 3. Results

### 3.1. Cough/Sneezing Scores, Antibiotic Use

Mean cough scores were 0.36 in group A and 0.27 in group B for cohort 1, 1.2 vs. 0.6 for cohort 2, and 2.0 vs. 2.6 for cohort 3. The respective sneezing scores were 6.3 vs. 3.8, 3.4 vs. 6.7, and 6.4 vs. 5.1. Significant differences were not found neither at the cohort level nor when comparing complete data sets for groups A and B. The animals had not to be treated by antibiotics besides the initial antibiosis against ileitis and arthritides throughout the whole study period.

### 3.2. ADWG, Final Weights, Losses

The weights of the measurements at week 15 and final weights at week 23 are summarized for all three cohorts of both vaccination groups in Table 1.

The mean overall ADWG_[inclusion–week 15]_ was 547.9 g and 547.2 g for groups A and B, respectively, and the mean ADWG_[week 15–week 23]_ was 813.5 g and 793.5 g, respectively. Overall ADWG_[inclusion–week 23]_ was 656.5 g for group A and 649.4 g for group B, which is a difference of 7.2 g. The mean final weights were 98.73 ± 9.13 kg and 97.73 ± 9.35 kg, respectively, which is a difference of 1 kg, without being statistically significant, however. Only at weighing time point 2 in week 15 was there a trend (*p* = 0.088) for higher weights in group B (52.50 ± 6.11 kg) vs. group A (51.67 ± 5.1 kg).

Losses due to euthanasia primarily because of wasting or Streptococci-induced arthritides were 3/111 (2.7%) in group A and 3/108 (2.8%) for group B in the first cohort, none at all in cohort 2, and 2/110 (1.8%) and 1/110 (0.9%) in cohort 3, respectively. These minimal numbers in both groups made a statistical testing impossible.

### 3.3. Lung Scores

In 2021, i.e., before start of the study, a CLP was performed (n = 33). The EP index was 1.88 with a prevalence of bronchopneumonic lungs (BP) of 75.76%. CP (cranial pleurisy) was evident in 27.27% of lungs, and 15.15% of lungs exhibited scars. The mean APP-index was 1.18.

**Figure 1:**
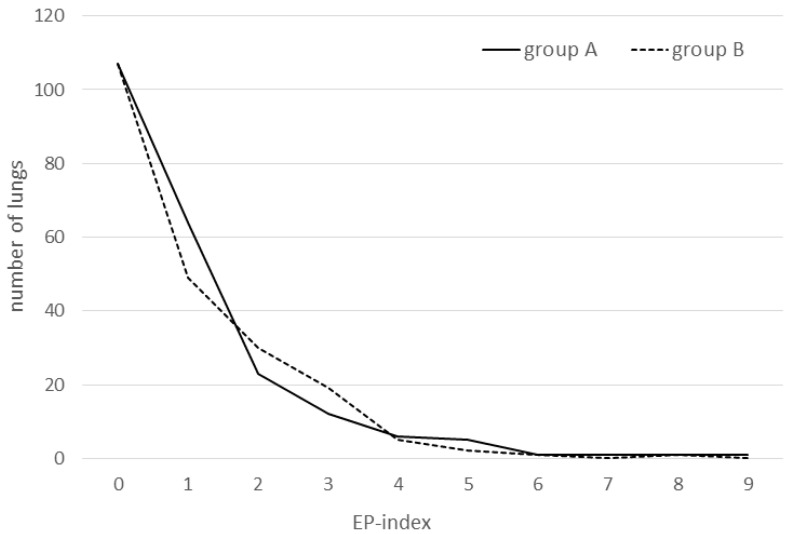
Distribution of EP index values over all three consecutive fattening cohorts in group A (IM vaccine, n = 221) and group B (ID vaccine, n = 214).

In the present study, not all pigs being weighed before slaughter could be scored for logistic reasons. No significant differences could be found between the respective score parameters of groups in the single cohorts or when analyzing entire groups A and B over the complete course of the trial. Only a trend towards a lower APP-index in group B within cohort 2 could be found (*p* = 0.064). The lung score data are summarized in Table 2 and the EP score distribution is shown in Figure 1.

## 4. Discussion

The best methods for prophylaxis against the occurrence of PRDC are hygiene, a good stable climate, the avoidance of overcrowding, and vaccination of pigs against *M.hp.* and PCV2, as these two pathogens are among the most prominent primary initiators of infectious lung diseases in commercial swine, at least in western and central Europe [1,10]. Therefore, veterinary pharma companies focusing on swine continuously work to optimize their vaccines against these two pathogens. This has led to the release of very potent inoculants, which are characterized by actual and strongly immunogenic epitopes as well as potent adjuvants. Additionally, there is a trend to create bi- or multivalent vaccines and to formulate them in a way so that they can be administered intradermally. The ID route has the advantage of being very well tolerated by the animals and stressing them only minimally. Additionally, there are no (or only minimal) risks of cross-contaminations and iatrogenic infections when working with needleless devices. However, the veterinarian or the farmer has to become accustomed to the handling of the ID device. When starting the trial, we had to initially revaccinate a small number of piglets due to failures in ID vaccine administration, which were evident due to the presence of liquid on the skin after vaccination instead of a circular area of dermal turgor at the vaccination site. However, with some experience, the vaccination procedure worked well afterwards.

From an epidemiological as well as clinical point of view, both vaccines improved and protected the herd’s lung health in an excellent manner, as reflected in the high daily weight gains, the lack of a need for antibiotic treatments, comparably low cough/sneezing indices, and well stabilized lung score parameters. The baseline EP index decreased by approximately 50% in both groups and the overall prevalence of bronchopneumonic lungs by about one third. Due to coupling of lung lesions for pathophysiological reasons [10,17], the APP-index also decreased by about 75%.

Even more interesting is the study of changes in lung score parameters over time, as analysis of dynamic processes is generally more meaningful than judging separate data. A good example is a field study investigating the responsiveness of lung health to specific vaccination in a pig herd suffering from infection with *Actinobacillus pleuropneumoniae* [17]. In the present case, values of all lung score parameters (with the exception of the APP-index) rose in both groups between cohorts 1 and 2, thus revealing that mycoplasmas again actively circulated at that time. However, from then on, i.e., during the time span between checking cohorts 2 and 3, group A parameters declined, whereas those of group B increased (with the exception of scars), which may be interpreted as a higher efficacy of the IM vaccine to stabilize and reduce the infection process during an active disease. Thus, if the trial had been prolonged, the small total score differences probably would have changed in favor of group A. However, the present median EP index of 1.0 was definitely lower compared with the median EP score of 2.0 in pigs vaccinated with different *M.hp.*-vaccines [10], emphasizing the necessity for only very cautiously interpreting or generalizing data obtained by field studies like this one. In aforementioned study, non-vaccinated pigs exhibited a median EP-score of 5.0, whereas here the initial EP-score was around 2. Maximum observable EP-score values as well as incidences of scars and cranial pleurisies were in the same and comparably quite low range in both groups when compared to data of other studies [10,12], thus also pointing towards a high efficacy to both vaccines tested.

Besides the benefits of slaughter-lung checks for monitoring the success of a vaccination measure, it is important to mention that the veterinarian should not rely primarily on positive PCR-results in the context of the clinical diagnosis of EP due to the weaknesses of this method concerning respiratory mycoplasmas, as is our own experience and was also shown in a recent study, where out of 790 piglets from 16 farms in Austria and Germany with a known history of EP, *M.hp.*-DNA was only detectable in 0.4% of tracheobronchial swabs [11]. Lung checks at slaughter in association with clinical observations (dry coughs, dyspnea) have more power in diagnosing EP than (only) molecular methods due to the high correlation of typical EP-like lesions with an infection with *M.hp.* [18].

As could be observed in our study and based on observations in other farms, piglets vaccinated intramuscularly with vaccine A stopped feeding for about one day and exhibited depressed behavior. This was reflected in higher weights in animals of group B at time point 2, i.e., at week 15, and was also already observed in another study comparing these two vaccines [19]. However, this difference was reversed at the time of slaughter, albeit not at a significant level.

One major, but inevitable limitation of this study is the lack of a control group. Although desirable from a scientific point of view, the inclusion of a control group was not feasible as this was a field trial. In view of the existing EP, the impact on the animals’ health and the farm enterprise of not vaccinating a group of piglets could not be justified.

Finally, it is worth mentioning that with the implementation of the new vaccines, no further antibiosis using tylosin-tartrate had been necessary, which is an additional indication of the quality of the two vaccines under investigation and the consequent vaccination management performed on that farm. In summary, also the non-inferiority of the ID vaccine could be demonstrated for this setting.

## 5. Conclusions

Both investigated vaccines proved highly effective in stabilizing lung health in the fattening farm investigated. However, it has to be considered that on other farms affected by respiratory disease and with different respiratory pathogens, the vaccines under investigation would probably exhibit different responses, which requires further investigation in field studies.

## Figures and Tables

**Table 1:** Weights (kg; mean and SD) at indicated time points for animals of group A (IM vaccine) and group B (ID vaccine).

Cohort	n		Week 15		n		Week 23	
	A	B	A	B	A	B	A	B
1	95	106	53.76 (6.80)	53.38 (5.72)	100	103	97.69 (9.46)	96.22 (9.02)
2	109	110	51.3 (5.97)	53.13 (4.78)	87	91	102.84 (9.27)	101.71 (8.83)
3	60	59	49.02 (3.64)	49.74 (3.16)	93	80	96.00 (8.64)	95.14 (10.21)

**Table 2:** Lung score data at slaughter for animals of group A (IM vaccine) and group B (ID vaccine). Index values are given as means and SD. EP: enzootic pneumonia, BP: bronchopneumonia, CP: cranial pleurisy, APP: *A.pp.*-pleuropneumonia.

Cohort	n		EP Index		BP (%)		CP (%)		Scars (%)		APP-Index	
	A	B	A	B	A	B	A	B	A	B	A	B
1	69	70	0.35 (0.66)	0.26 (0.70)	26.09	15.71	20.29	18.57	18.84	5.71	0.32 (0.80)	0.41 (0.94)
2	59	48	1.66 (1.86)	1.19 (1.16)	71.19	64.58	28.81	20.83	22.03	25.00	0.36 (1.01)	0.06 (0.43)
3	93	96	1.12 (1.47)	1.31 (1.49)	58.06	67.71	20.43	22.92	19.35	22.92	0.26 (0.67)	0.29 (0.75)
Total	221	214	1.02 (1.48)	0.94 (1.31)	51.58	48.42	22.62	21.03	19.91	17.76	0.30 (0.81)	0.28 (0.78)

## Data Availability

Not applicable.
